# Lifestyle and subsequent meningioma in childhood cancer survivors: A report from the St. Jude Lifetime Cohort study

**DOI:** 10.1002/cnr2.1944

**Published:** 2023-11-27

**Authors:** Aron Onerup, Sedigheh Mirzaei S., Shalini Bhatia, Megan E. Ware, Lenat Joffe, Lucie M. Turcotte, Chelsea G. Goodenough, Yadav Sapkota, Stephanie B. Dixon, Matthew D. Wogksch, Matthew J. Ehrhardt, Gregory T. Armstrong, Melissa M. Hudson, Kirsten K. Ness

**Affiliations:** ^1^ Department of Epidemiology and Cancer Control St Jude Children's Research Hospital Memphis Tennessee USA; ^2^ Department of Pediatrics Institute of Clinical Sciences, Sahlgrenska Academy, University of Gothenburg Gothenburg Sweden; ^3^ Department of Biostatistics St Jude Children's Research Hospital Memphis Tennessee USA; ^4^ Department of Pediatrics Zucker School of Medicine at Hofstra/Northwell New Hyde Park New York USA; ^5^ Department of Pediatrics University of Minnesota Minneapolis Minnesota USA; ^6^ Department of Oncology St Jude Children's Research Hospital Memphis Tennessee USA

**Keywords:** body composition, childhood cancer, epidemiology, fitness, meningioma, survivorship

## Abstract

**Background:**

Lifestyle is associated with meningioma risk in the general population.

**Aims:**

We assessed longitudinal associations between lifestyle‐associated factors and subsequent meningiomas in childhood cancer survivors.

**Methods and results:**

Childhood cancer survivors age ≥18 years in the St. Jude Lifetime Cohort Study were evaluated for body composition, self‐reported physical activity, cardiopulmonary fitness, muscle strength, smoking, and alcohol consumption at baseline. Time to first meningioma analyses were performed, adjusted for sex, age at diagnosis and baseline assessment, treatment decade, and childhood cancer treatment exposures. The study included 4,072 survivors (47% female; [mean (SD)] 9 (6) years at diagnosis; 30 (8.5) years at the start of follow‐up, with 7.0 (3.3) years of follow‐up). 30% of the participants were survivors of acute lymphoblastic leukemia and 29% of the participants had received cranial radiation. During follow‐up, 90 participants developed ≥1 meningioma, of whom 73% were survivors of acute lymphoblastic leukemia, with cranial radiation being the strongest risk factor (relative risk [RR] 29.7, 95% confidence interval [CI] 10.6‐83.2). Muscle strength assessed by knee extension was associated with a lower risk of developing a meningioma in the adjusted analyses (RR 0.5, 95% CI 0.2‐1.0, *p* = 0.04 for quartiles 3‐4 vs. 1). No other lifestyle‐associated variable was associated with subsequent meningioma.

**Conclusion:**

Independent of cranial radiation, muscle strength was associated with a lower risk of developing a subsequent meningioma in childhood cancer survivors.

## 1 BACKGROUND

Low physical activity and increased body fat are associated with a higher risk of developing meningioma in the general population,^1^, ^2^ in addition to affecting women more than men.^3^ Childhood cancer survivors are at increased risk of developing meningioma, attributed to cranial radiotherapy.^4^ These meningiomas generally occur decades after the childhood cancer diagnosis and may cause both neurological sequelae and death.^4^ Whether lifestyle can modify the increased risk of meningioma in childhood cancer survivors is not known. This study aimed to assess longitudinal associations between lifestyle and subsequent meningiomas in childhood cancer survivors included in the St Jude Lifetime Cohort study (SJLIFE).^5^

## 2 METHODS

This study was an observational analysis within SJLIFE. The assessments in SJLIFE have been described previously.^5^, ^6^ Participants enrolled in the St. Jude Lifetime Cohort who were ≥18 years of age and ≥5 years from childhood cancer diagnosis at the time of assessment, and had undergone physical performance and/or body composition assessment were eligible for this study.

### 2.1 Exposures

#### 2.1.1 Cardiopulmonary fitness

Cardiopulmonary fitness was evaluated by meters walked from the 6‐min walk test.^7^, ^8^, ^9^ The results were categorized into quartiles for the analyses and the lowest quartile was used as reference in the statistical analyses.

#### 2.1.2 Muscular strength

Muscular strength was evaluated by knee extension strength (Nm/kg) and handgrip strength testing (kg). Isokinetic knee extension strength was measured as peak torque from five repetitions at 60° per second. The maximum value was used for analyzing both knee extension strength and handgrip strength. The results were categorized into quartiles, with the third and fourth quartiles combined. The lowest quartile was used as reference in the analyses.

#### 2.1.3 Body composition

Body composition was evaluated with body mass index (BMI; kg/m^2^) from the clinical evaluation and body fat percentage from DEXA evaluations. We categorized BMI as underweight (<18.5), normal (≥18.5 and <25), overweight (≥25 and <30), and obesity (≥30). Body fat percentage was categorized according to the Obesity Medicine Association 2022 guidelines.^10^ For men, 25%–29% was categorized as pre‐obesity and ≥30% as obesity. For women, 30%–34% was categorized as pre‐obesity and ≥35% as obesity. Normal weight was used as reference for both BMI‐ and DEXA‐defined variables.

#### 2.1.4 Physical activity

Physical activity was self‐reported as moderate‐to‐vigorous physical activity minutes per day, which was then converted into metabolic equivalent task (MET) hours per week and categorized into 0–3, 3–6, and ≥6 METh/week. 0–3 MET hours per week was used as reference.

#### 2.1.5 Smoking

Self‐reported smoking, categorized as never, previous, or current smoking. Current smoking was used as reference in the analyses.

#### 2.1.6 Alcohol consumption

Risky drinking was defined as >3 drinks per day or >7 per week for women, >4 per day or >14 per week for men. Risky drinking was used as reference in the analyses.

### 2.2 Outcome

Time to first meningioma during survivorship in adult age was the outcome, including both benign and malignant meningiomas. Only meningiomas occurring after the baseline assessment were included. All SJLIFE participants have ascertained follow‐up of subsequent malignancies through medical record abstraction and yearly follow‐up with the St Jude Cancer Registry.^11^ There is no routine CNS‐imaging included in the research evaluations in SJLIFE. Hence, all meningiomas were diagnosed from clinically motivated follow‐up. Meningioma diagnoses were ascertained from histopathological diagnosis where available and otherwise from imaging.

### 2.3 Treatment exposures

Medical record abstraction for eligible SJLIFE participants includes abstraction of all chemotherapy received, including cumulative doses.^11^ In this study, we included dichotomized treatment exposures (yes/no) to anthracyclines, alkylating agents, epipodophyllotoxins, platinum, and brain radiation.

### 2.4 Statistical methods

Piecewise exponential models estimated risk for meningioma as relative risk (RR) with 95% confidence interval (CI) adjusted for sex, age and calendar year at childhood cancer diagnosis, age at baseline assessment, chemotherapy exposures (alkylating agents, platinum, epipodophyllotoxins, anthracyclines), and cranial radiation. *P*‐values from the piecewise exponential models for each comparison are provided. Follow‐up started 1 year after the baseline campus visit and stopped at the time of first meningioma, date of last contact, or death, whichever came first. Each model included one of the exposures of interest (cardiopulmonary fitness, body composition, physical activity, strength, smoking, or risky drinking) and the predefined confounders known to affect the risk for meningiomas.

## 3 RESULTS

The study included 4072 survivors, 1925 (47%) female, 1133 (29%) previous/current smokers, 1435 (37%) risky drinkers, 1384 (34%) with obesity; mean (standard deviation [SD]) age was 8.7 (5.7) years at diagnosis, and 30 (8.4) years at baseline SJLIFE evaluation (Table 1). Subsequent length of follow‐up was 7.1 (3.3) years after assessment. At least one meningioma was experienced by 90 (2.2%) survivors, of whom 66 (73%) had been treated for acute lymphoblastic leukemia (ALL), and 85 (96%) had received cranial radiation therapy (Table 1).

**TABLE 1:** Demographic, lifestyle, diagnosis, and treatment variables according to body composition.

Characteristic	Developed meningioma	No meningioma	Total
Number of participants	90	3982	4072
Sex, female	50 (56%)	1875 (47%)	1925 (47%)
Age at baseline, years, mean (SD)	36.2 (7.7)	30.0 (8.4)	30.1 (8.5)
Years of follow‐up, mean (SD)	4.5 (2.7)	7.1 (3.3)	7.0 (3.3)
Race/ethnicity			
Non‐Hispanic Caucasian	82 (91%)	3175 (80%)	3257 (80%)
Non‐Hispanic African American	8 (9%)	615 (15%)	623 (15%)
Hispanic	0 (0%)	114 (3%)	114 (3%)
Other	0 (0%)	78 (2%)	78 (2%)
Educational attainment			
Up to high school or GED	29 (33%)	1164 (31%)	1193 (31%)
Training after high school or some college	31 (35%)	1345 (36%)	1376 (36%)
College graduate or postgraduate	28 (32%)	1206 (32%)	1234 (32%)
Missing	2	267	269
Household income, per year			
<$20 000	35 (50%)	1357 (43%)	1392 (43%)
$20 000–<$59 999	19 (27%)	1034 (33%)	1053 (33%)
≥ <$60 000	16 (23%)	747 (24%)	763 (24%)
Missing	20	844	864
Body mass index, kg/m^2^, mean (SD)	32.4 (9.8)	28.1 (7.1)	28.2 (7.2)
Underweight	2 (2%)	148 (4%)	150 (4%)
Normal weight	17 (19%)	1388 (35%)	1405 (35%)
Overweight	26 (29%)	1106 (28%)	1132 (28%)
Obesity	45 (50%)	1339 (34%)	1384 (34%)
Body fat percentage, %, mean (SD)	36 (8)	32 (9)	32 (9)
Non obese	5 (12%)	899 (28%)	904 (28%)
Pre obesity	5 (12%)	744 (23%)	749 (23%)
Obesity	31 (76%)	1576 (49%)	1607 (49%)
Missing	49	763	812
Cardiorespiratory fitness			
6‐min walk test, meters walked, mean (SD)	523 (106)	556 (104)	556 (104)
Missing	4	159	163
Grip strength, kg, mean (SD)	34 (12)	39 (13)	39 (13)
Missing	0	8	8
Knee extension, Nm/kg, mean (SD)	120 (57)	142 (56)	141 (56)
Missing	11	449	460
Self‐reported physical activity, METh/week, median (IQR)	6.0 (0.0–19.1)	9.0 (0.0–24.0)	9.0 (0.0–24.0)
Missing	4	235	239
Smoking history			
Never	69 (79%)	2715 (71%)	2784 (71%)
Past	11 (13%)	453 (12%)	464 (12%)
Current	7 (8%)	662 (17%)	669 (17%)
Missing	3	152	155
Heavy/risky drinking of alcohol	18 (21%)	1417 (38%)	1435 (37%)
Missing	4	220	224
Healthy lifestyle score			
Poor	41 (46%)	1905 (48%)	1946 (48%)
Moderate	36 (40%)	1311 (33%)	1347 (33%)
Healthy	13 (14%)	766 (19%)	779 (19%)
Decade of primary cancer diagnosis			
1960–1970	8 (9%)	111 (3%)	119 (3%)
1971–1980	42 (47%)	583 (15%)	625 (15%)
1981–1990	28 (31%)	1064 (27%)	1092 (27%)
1991–2000	11 (12%)	1346 (34%)	1357 (33%)
2001	1 (1%)	878 (22%)	879 (22%)
Primary childhood cancer diagnosis			
ALL	66 (73%)	1140 (29%)	1206 (30%)
Central nervous system tumor	12 (13%)	541 (14%)	553 (14%)
Other	12 (13%)	2301 (57%)	2313 (57%)
Age at primary cancer diagnosis, years	7 (5)	9 (6)	9 (6)
Cancer treatment			
Received cranial radiation	85 (96%)	1088 (28%)	1173 (29%)
Received alkylating agents	54 (60%)	2305 (58%)	2359 (58%)
Received anthracyclines	41 (46%)	2303 (58%)	2344 (58%)
Received platinum	10 (11%)	559 (14%)	569 (14%)
Received epipodophyllotoxins	41 (46%)	1422 (36%)	1463 (36%)
Hematopoietic stem cell transplant	6 (7%)	319 (8%)	325 (8%)

*Note*: Numbers are *n* (%) if not stated otherwise.

Abbreviation: MET, metabolic equivalent task.

### 3.1 Univariate analyses

In the univariate analyses, self‐reported physical activity was not associated with the subsequent risk of developing meningioma (RR 0.7, 95% CI 0.5–1.2 for high vs. low categories), while both BMI‐ (RR 2.5, 95% CI 1.5–4.4 for obesity vs. normal weight) and DEXA‐assessed (RR 6.3, 95% CI 2.5–15.6 for obesity vs. normal weight) obesity were associated with an increased risk. Cardiorespiratory fitness (RR 0.4, 95% CI 0.2–0.7 for high vs. low quartiles), grip strength (RR 0.5, 95% CI 0.3–0.7 for quartiles 3–4 vs. 1), knee extension strength (RR 0.3, 95% CI 0.2–0.5 for quartiles 3–4 vs. 1), smoking (RR 1.8, 95% CI 1.1–3.1 for never smoking vs. current smoking) and risky drinking (RR 2.3, 95% CI 1.4–3.9 for no risky drinking vs. risky drinking) were associated with lower risk of developing a meningioma (Table 2).

**TABLE 2:** Hazard rate ratios for associations between lifestyle‐associated variables and meningioma.

Comparison	Univariate analyses		Multivariable analyses	
	RR (95% CI)	*P* for comparison	RR (95% CI)	*P* for comparison
Body mass index				
Overweight (25–29.9) vs. normal	1.8 (1.0–3.3)	0.06	1.3 (0.7–2.4)	0.42
Obesity (≥30) vs. normal	2.5 (1.5–4.4)	0.001	1.2 (0.7–2.1)	0.59
Body fat percentage				
Pre‐obesity vs. normal	2.4 (0.8–6.9)	0.11	1.1 (0.4–3.3)	0.81
Obesity vs. normal	6.3 (2.5–15.6)	<0.001	1.8 (0.7–4.7)	0.23
Self‐reported physical activity				
3–6 vs. 0–3 METh/week	0.7 (0.4–1.3)	0.26	NA	NA
≥6 vs. 0–3 METh/week	0.7 (0.5–1.2)	0.20	NA	NA
6‐min walk test				
Quartile 2 vs. 1	0.6 (0.4–1.1)	0.09	0.8 (0.5–1.5)	0.53
Quartile 3 vs. 1	0.5 (0.3–0.9)	0.02	0.8 (0.4–1.4)	0.43
Quartile 4 vs. 1	0.4 (0.2–0.7)	0.002	0.7 (0.4–1.4)	0.33
Grip strength				
Moderate vs. low	0.4 (0.2–0.7)	0.002	0.6 (0.3–1.1)	0.12
High vs. low	0.5 (0.3–0.7)	0.001	0.8 (0.3–1.7)	0.49
Knee extension				
Moderate vs. low	0.5 (0.3–0.9)	0.03	0.7 (0.4–1.1)	0.14
High vs. low	0.3 (0.2–0.5)	<0.001	0.5 (0.2–1.0)	0.04
Never smoker	1.8 (1.1–3.1)	0.02	1.6 (0.9–2.9)	0.11
Absence of risky drinking	2.3 (1.4–3.9)	0.002	1.3 (0.7–2.3)	0.36

*Note*: One lifestyle variable was included in each model. All models were adjusted for sex, age at diagnosis and baseline assessment, treatment decade, and childhood cancer treatment exposures.

Abbreviations: BMI, body mass index; CNS, central nervous system; MET, metabolic equivalent task; RR, relative risk.

### 3.2 Multivariable models

As expected, cranial radiation exposure was the strongest risk factor for developing meningiomas in this childhood cancer survivor population (RR 29.7, 95% CI 10.6–83.2). In our multivariable models including treatment variables, knee extension strength was the only lifestyle variable associated with the risk of developing a subsequent meningioma (RR 0.5, 95% CI 0.2–1.0 for quartiles 3–4 vs. 1, Table 2). None of the meningiomas occurred in participants with growth hormone replacement at baseline or neurofibromatosis types 1 or 2. For BMI‐ (RR 1.2, 95% CI 0.7–2.1) and fat percentage‐defined (RR 1.8, 95% CI 0.7–4.7) obesity, the adjusted estimates changed considerably from the univariate analyses, indicating confounding by treatment exposures. For cardiorespiratory fitness (RR 0.7, 95% CI 0.4–1.4) and grip strength (RR 0.8, 95% CI 0.3–1.7), the difference in estimates between univariate and adjusted analyses was less dramatic, albeit there were no associations.

## 4 DISCUSSION

In this observational study of 4072 childhood cancer survivors, we report a protective association between knee extension strength and the risk of developing meningioma, independent of cranial radiation therapy. Previous studies have reported increased risk of meningioma with increasing body fat and physical inactivity in the general population.^1^, ^2^

Our study could not confirm associations between self‐reported physical activity or BMI and meningioma. There are several possible explanations for this difference. Childhood cancer survivors are at increased risk of sarcopenic obesity and BMI is a poor measure for identifying obesity in childhood cancer survivors, compared to DEXA.^12^ This might reflect the difference in RR between BMI‐ and DEXA‐assessed obesity, albeit none of them were significant after adjustment for treatment exposures and other potential confounders. Self‐reported physical activity measures have relatively low sensitivity in the general population.^13^ Objectively assessed cardiorespiratory and muscular fitness are more sensitive measures and are improved mainly by exercise training and less by low‐intensity PA,^14^ and have been reported to be stronger predictors of other health outcomes, such as all‐cause mortality.^14^ A recently published study showed associations between cardiorespiratory fitness and site‐specific cancers across several organ systems but did not look at meningiomas specifically.^15^ In our univariate analyses, both favorable body composition and all measures of cardiorespiratory and musculoskeletal fitness were associated with decreased risk of developing meningioma. Our multivariable analyses could only confirm associations for knee extension strength. This might be explained by residual confounding from cranial radiation treatment but may also reflect true associations between, for example, exercise and meningiomas. This should be further assessed in studies from other cohorts and in future studies with longer follow‐up. Since cranial radiation increases the risk of both meningioma and obesity, it can be hard to discriminate possible direct effects of obesity on meningioma. It is also possible that some of the results, for example, for DEXA‐assessed body composition suffered from low statistical power. Most survivors who developed a meningioma did not undergo DEXA scans and a longer follow‐up might result in significant associations. The fact that all associations for body composition and fitness were in the expected direction, albeit not significant, could imply that a longer follow‐up with more meningiomas during follow‐up would yield further significant associations. There is a long‐time latency between childhood cancer treatment and meningioma onset. Our population was followed from a mean of 22–28 years after diagnosis, corresponding to the peak in meningioma incidence in other studies.^4^

This study was performed within SJLIFE, with clinically ascertained health outcomes and objectively assessed fitness and body composition. To our knowledge, our study is the first observational cohort study assessing the association between lifestyle factors and meningioma in childhood cancer survivors. The main limitation is the relatively short follow‐up of participants, resulting in a limited number of events, limiting statistical power. The absence of histopathological diagnosis for the majority of meningiomas is a limitation. However, performing neurosurgical operations to obtain tissue samples when not clinically motivated cannot be done. The absence of systematically performed CNS imaging in all participants in SJLIFE is a limitation. However, this would have either exposed participants to unnecessary radiation from CT scans or required them to undergo MRI scans and would need to be justified. These results from a single observational study need to be confirmed in other populations, preferably with diverse ethnicities or from different nations, to strengthen any assumptions on causality. However, they are supported by similar associations in the general population.

In conclusion, independent of treatment exposures, knee extension strength was associated with a lower risk of developing meningioma in childhood cancer survivors. It remains to be shown whether this reflects a causal effect.

## AUTHOR CONTRIBUTIONS

**Aron Onerup:** Conceptualization (lead); funding acquisition (supporting); investigation (equal); methodology (equal); writing – original draft (lead); writing – review and editing (lead). **Sedigheh Mirzaei S.:** Conceptualization (equal); formal analysis (equal); investigation (equal); methodology (equal); supervision (equal); validation (equal); writing – review and editing (equal). **Shalini Bhatia:** Formal analysis (lead); investigation (equal); methodology (supporting); validation (equal); writing – review and editing (equal). **Megan E. Ware:** Investigation (supporting); methodology (supporting); writing – review and editing (supporting). **Lenat Joffe:** Investigation (supporting); methodology (supporting); writing – review and editing (supporting). **Lucie M. Turcotte:** Investigation (supporting); methodology (supporting); writing – review and editing (supporting). **Chelsea G. Goodenough:** Investigation (supporting); methodology (supporting); writing – review and editing (supporting). **Yadav Sapkota:** Investigation (supporting); methodology (supporting); writing – review and editing (supporting). **Stephanie B. Dixon:** Conceptualization (supporting); investigation (equal); methodology (supporting); writing – review and editing (equal). **Matthew D. Wogksch:** Investigation (supporting); methodology (supporting); writing – review and editing (supporting). **Matthew J. Ehrhardt:** Conceptualization (supporting); investigation (supporting); methodology (supporting); writing – review and editing (supporting). **Gregory T. Armstrong:** Conceptualization (supporting); investigation (supporting); methodology (supporting); project administration (equal); resources (equal); writing – review and editing (supporting). **Melissa M. Hudson:** Conceptualization (supporting); funding acquisition (equal); investigation (supporting); methodology (supporting); resources (supporting); writing – review and editing (supporting). **Kirsten K. Ness:** Conceptualization (equal); funding acquisition (equal); investigation (equal); methodology (equal); project administration (equal); resources (equal); supervision (lead); writing – review and editing (equal).

## CONFLICT OF INTEREST STATEMENT

The authors have stated explicitly that there are no conflicts of interest in connection with this article.

## ETHICS STATEMENT

The St. Jude Lifetime Cohort study was approved by the Institutional Review Board at St. Jude Children's Research Hospital, with current approval date December 3, 2023.

## Data Availability

The data supporting this study's findings are openly available at: https://www.stjude.cloud/research-domains/cancer-survivorship. Data specific to this paper will be uploaded to https://zenodo.org concomitant with the publication of the manuscript.
